# Wetting Ridge‐Guided Directional Water Self‐Transport

**DOI:** 10.1002/advs.202204891

**Published:** 2022-10-17

**Authors:** Lingxiao Wang, Kai Yin, Qinwen Deng, Qiaoqiao Huang, Jun He, Ji‐An Duan

**Affiliations:** ^1^ Hunan Key Laboratory of Nanophotonics and Devices School of Physics and Electronics Central South University Changsha 410083 P. R. China; ^2^ The State Key Laboratory of High Performance and Complex Manufacturing College of Mechanical and Electrical Engineering Central South University Changsha 410083 P. R. China

**Keywords:** directional water self‐transport, femtosecond laser, heterogeneous superwettability, lubricant infusion, wetting ridge

## Abstract

Directional water self‐transport plays a crucial role in diverse applications such as biosensing and water harvesting. Despite extensive progress, current strategies for directional water self‐transport are restricted to a short self‐driving distance, single function, and complicated fabrication methods. Here, a lubricant‐infused heterogeneous superwettability surface (LIHSS) for directional water self‐transport is proposed on polyimide (PI) film through femtosecond laser direct writing and lubricant infusion. By tuning the parameters of the femtosecond laser, the wettability of PI film can be transformed into superhydrophobic or superhydrophilic. After trapping water droplets on the superhydrophilic surface and depositing excess lubricant, the asymmetrical wetting ridge drives water droplets by an attractive capillary force on the LIHSS. Notably, the maximum droplet self‐driving distance can approach ≈3 mm, which is nearly twice as long as the previously reported strategies for direction water self‐transport. Significantly, it is demonstrated that this strategy makes it possible to achieve water self‐transport, anti‐gravity pumping, and chemical microreaction on a tilted LIHSS. This work provides an efficient method to fabricate a promising platform for realizing directional water self‐transport.

## Graphical abstract

Inspired by nature, a strategy for directional water self‐transport is proposed on a lubricant‐infused heterogeneous superwettability surface, which is fabricated via femtosecond laser direct writing and lubricant infusion. The water droplet on the sample surface can be transported to the trapped droplet by an asymmetrical wetting ridge. This work extends the applications of multifunctional slippery surfaces and directional water self‐transport.

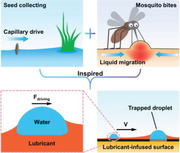

## 1 Introduction

Directional liquid transport has attracted significant attention on account of its tremendous application potential ranging from liquid collection,^[^ ^1^, ^2^, ^3^ ^]^ microfluidic operation,^[^ ^4^, ^5^ ^]^ chemical microreaction,^[^ ^6^ ^]^ and biomedical application.^[^ ^7^ ^]^ So far, many researchers have used external energy input, including magnetism,^[^ ^8^, ^9^ ^]^ electric field,^[^ ^10^ ^]^ or light signals,^[^ ^11^, ^12^, ^13^ ^]^ as an active driving force to achieve efficient directional liquid transport, which typically requires doping of characteristic nanoparticles and multistep fabrication technology. In nature, lots of creatures have evolved unique microscaled structures with the ability to spontaneous and directional liquid transport.^[^ ^14^, ^15^, ^16^, ^17^, ^18^, ^19^ ^]^ For instance, the Araucaria leaf, which is composed of periodically arranged 3D ratchets with transverse and longitudinal reentrant curvatures, allows the fluids with different surface tension to spread along different directions.^[^ ^20^ ^]^ Namib desert beetle can harvest water droplets from the fog by using their heterogeneous wettability structured back that consists of wax‐coated hydrophobic regions and non‐waxy hydrophilic bumps.^[^ ^21^ ^]^ Learning from successful directional liquid transport strategies of natural organisms, several structured surfaces with asymmetric geometry or gradient wettability have been developed to achieve spontaneous and directional liquid transport.^[^ ^22^, ^23^, ^24^, ^25^ ^]^ These designed functional surfaces can generate a passive driving force to steer the liquids, which exhibits greater application prospects.

More recently, inspired by the surface of Nepenthes pitcher plants, lubricant‐infused porous surface (LIPS) has great potential to be used as an excellent platform for water droplets transport due to its unique slippery properties.^[^ ^26^, ^27^, ^28^, ^29^, ^30^, ^31^, ^32^, ^33^ ^]^ On the basis of ultralow adhesion force between water and lubricant, water droplets can be easily driven under a small driving force. Great efforts have been devoted to the realization of directional water self‐transport on the LIPS. For example, Zhao et al. developed a multi‐bioinspired slippery surface with wettable hollow bump arrays by colloidal self‐assembly, photolithography, and mold replication, which was successfully applied for directional droplet pumping and water harvesting.^[^ ^34^ ^]^ Yao et al. designed a silicone oil‐infused polydimethylsiloxane with surface‐piercing hydrogel dots via a liquid patterning method, which was competent for droplet condensation and sensitive biomedical analysis.^[^ ^35^ ^]^ These studies have extensively deepened the research of directional water self‐transport on the LIPS.^[^ ^36^, ^37^ ^]^ However, they still have several inherent limitations, such as a short self‐driving distance, single function, and complicated fabrication methods. Hence, it is an urgent need to develop a multifunctional slippery surface for achieving directional water self‐transport with a longer distance, and yet, benefiting from highly efficient fabrication.

Herein, we report a lubricant‐infused heterogeneous superwettability surface (LIHSS) on polyimide (PI) film fabricated via femtosecond laser direct writing and lubricant infusion, which enables asymmetric lubricant wetting ridge‐guided directional water self‐transport. Through twice femtosecond laser line‐by‐line scanning in different parameters, superhydrophobic (a contact angle of ≈155.6°) surface and superhydrophilic (a contact angle of ≈2.4°) surface are prepared on PI film, respectively. By virtue of the strong adhesion from superhydrophilicity, water droplets can be trapped on the superhydrophilic surface (SHLS) surrounded by a superhydrophobic surface (SHBS). Subsequently, excess lubricant is deposited on the heterogeneous superwettability surface to construct LIHSS. Water droplets released on the LIHSS are propelled under an attractive capillary force induced by lubricant wetting ridge, which can realize a maximum droplet self‐driving distance of ≈3 mm. Furthermore, we exhibit water self‐transport, anti‐gravity pumping, and chemical microreaction on the LIHSS with a small tilting angle, demonstrating its great potential in the fields of droplet manipulation.

## 2 Results and Discussion

**Figure 1:**
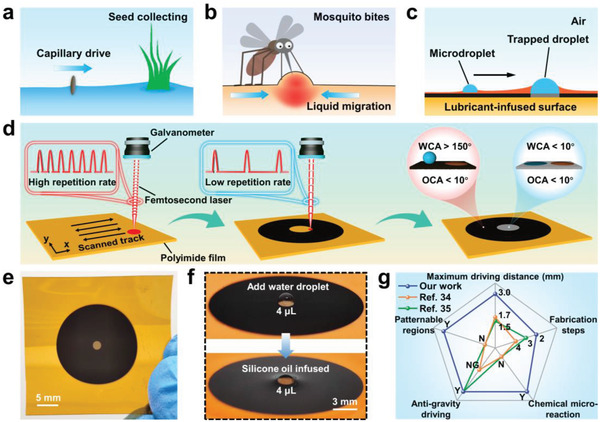
Preparation of lubricant‐infused heterogeneous superwettability surface. a) Emergent aquatic vegetation collecting seeds by capillary drive. b) Mosquito bites‐induced bulge structures on the human skin surface. c) Directional self‐transport of microdroplets on the lubricant‐infused heterogeneous superwettability surface. d) Schematic illustration of the fabrication steps for the heterogeneous superwettability surface on PI film. e) Optical photo of as‐prepared heterogeneous superwettability surface. f) Optical photos of heterogeneous superwettability surface after adding water droplet and silicone oil infusion. g) Comparison of the comprehensive performance for our directional water self‐transport strategy with previously reported strategies.

As we all know, emergent aquatic vegetation can collect seeds through water meniscus‐induced capillary force to realize seed dispersal (**Figure** **1**a). After mosquito bites, the surface of human skin will form a bulge via anaphylaxis and liquid migration (Figure 1b). Nepenthes pitcher plants employ a unique strategy to capture insects by using their slippery surfaces. Drawing inspiration from emergent aquatic vegetation, mosquito bites‐induced bulge, and Nepenthes pitcher plants, we put forward a LIHSS for directional water self‐transport. As shown in Figure 1c, the trapped droplet is tethered to the superhydrophilic area, while the microdroplet is on the lubricant‐infused superhydrophobic area and moves toward the direction of the trapped droplet under a capillary force.

To simultaneously fabricate superhydrophobicity and superhydrophilicity on the PI film, femtosecond laser direct writing technology is adopted in this experiment. Recently, this technology has been widely used in the field of regulating surface wettability on account of its exceptional advantages such as high precision, no‐contact processing, and high efficiency.^[^ ^38^, ^39^, ^40^, ^41^, ^42^, ^43^, ^44^, ^45^, ^46^ ^]^ Figure 1d demonstrates a schematic process for preparing a heterogeneous superwettability surface on the PI film. First, to guarantee uniformity and symmetry in every direction, a high repetition rate (200 kHz) and high power (700 mW) femtosecond laser was employed to scribe an annular superhydrophobic pattern through line‐by‐line scanning on the PI film. Then, considering the spreading behavior of a water droplet on the sample surface, we used a low repetition rate (5 kHz) and low power (40 mW) femtosecond laser to scribe a superhydrophilic circular pattern and finally obtained a heterogeneous superwettability surface. Besides, the maximum temperatures of the two scanning processes were completely different, indicating divergent femtosecond laser thermal accumulation effects (Figure S1, Supporting Information).^[^ ^47^ ^]^ Especially, the SHBS exhibited an ultrahigh water contact angle (WCA) and an ultralow oil contact angle (OCA), whereas both the WCA and OCA of the SHLS were extremely low. From the perspective of function, the circular superhydrophilic area was used to trap and fix a water droplet; the annular superhydrophobic area was a place where the microdroplet moves and slides after lubricant infusion. The ultralow OCA of the superhydrophobic area was conducive to the spread of lubricant and the construction of lubricant film on the sample surface. Meanwhile, different volumes of water droplets could be trapped on the obtained heterogeneous superwettability surface and display different macroscopic shapes, which further affect the maximum self‐driving distance. The high repetition rate and high power femtosecond laser‐treated PI (HRHPP) surface exhibited a dark appearance and strong solar absorptivity, while the low repetition rate and low power femtosecond laser‐treated PI (LRLPP) surface manifested with a grey appearance and low solar absorptivity (Figures S2 and S3, Supporting Information). Figure 1e gives an optical photo of the obtained heterogeneous superwettability surface, which consists of a circular SHLS and an annular SHBS. Because of adhesion force, a water droplet (≈4 µL) from a microinjector was confined to the circular SHLS. Finally, silicone oil was selected as the lubricant and added to the surface to form a silicone oil‐infused heterogeneous superwettability surface (Figure 1f). Compared with other reported methods, our strategy demonstrates huge advantages involving maximum driving distance, fabrication steps, chemical microreaction, anti‐gravity driving, and patternable regions (Figure 1g).

**Figure 2:**
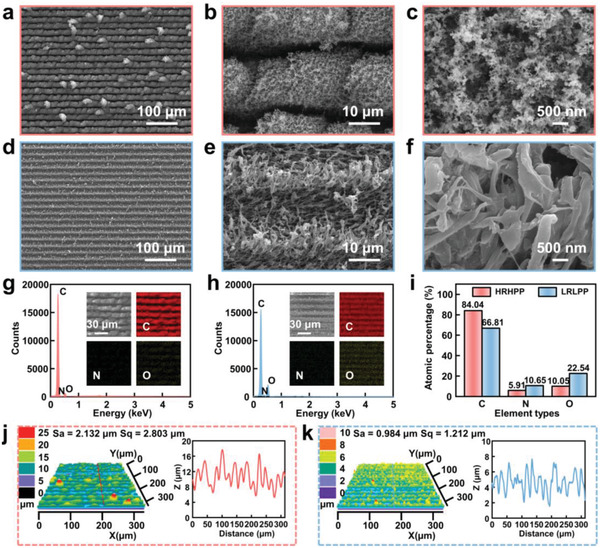
Surface morphology and chemical composition characterizations of PI films after femtosecond laser treatment. a–c) SEM images of the HRHPP surface at different magnifications. d–f) SEM images of the LRLPP surface at different magnifications. g,h) EDS spectra and elemental mapping images of C, N, and O for the HRHPP and LRLPP surfaces, respectively. i) Comparison of chemical compositions of the HRHPP and LRLPP surfaces. j,k) LCM 3D confocal images and cross‐sectional profiles of the HRHPP and LRLPP surfaces.

In order to get a better understanding of superwettability, we observed the surface morphologies and chemical compositions of the samples. **Figure** **2**a–f shows the scanning electron microscope (SEM) images of HRHPP and LRLPP surfaces under different magnifications. Compared with the flat and smooth surface of PI film (Figure S4, Supporting Information), both the HRHPP and LRLPP surfaces are rough and exhibit porous structures, contributing to lubricant diffusion on the surface. In particular, Figure 2a displays that the HRHPP surface is consist of group floc‐like microstructures and protrusions. High‐magnification SEM images of HRHPP surface show that the microstructures and protrusions are covered with nanoparticles, resulting from femtosecond laser strong thermal accumulation effects and ablated fragments redeposition (Figure 2b,c and Figure S5, Supporting Information). By contrast, Figure 2d–f shows that the LRLLP surface is covered by unevenly shaped arranged filiform microstructures, which is due to partial chain scission of the PI. Moreover, the SEM images of five different locations on HRHPP and LRLPP surfaces demonstrate that laser‐induced rough porous structures are uniformly distributed on the entire sample surface (Figures S6 and S7, Supporting Information). As can be seen from panels (g) and (h) of Figure 2, after femtosecond laser treatment, the chemical compositions of HRHPP and LRLPP surfaces are different, but the elements are evenly distributed on the surfaces. More concretely, for the HRHPP surface, the content of the C element apparently increases from 68.76% to 84.04%, the N element decreases from 9.05% to 5.91%, and the O element obviously decreases from 22.09% to 10.05%, revealing that it was seriously carbonized. However, the C, N, and O elements of LRLPP surfaces only change slightly in contrast to the elements of the pristine PI film surface (Figure 2i and Figure S4, Supporting Information). Furthermore, the 3D confocal image and surface roughness of the sample surface was investigated by laser confocal microscopy (LCM). As shown in Figure 2j, the LCM image of the HRHPP surface shows uniformly distributed microstructures with a depth between 5–18 µm, which is consistent with its SEM images. Correspondingly, the arithmetical mean roughness (Sa) and root mean square roughness (Sq) are 2.132 and 2.803 µm, respectively. Figure 2k indicates that the microstructures with a depth between 2–7 µm, Sa of 0.984 µm, and Sq of 1.212 µm are detected on the LRLPP surface, demonstrating a lower surface roughness. Besides, the LCM and SEM images of the boundary line between the SHLS and SHBS are shown in Figure S8, Supporting Information.

**Figure 3:**
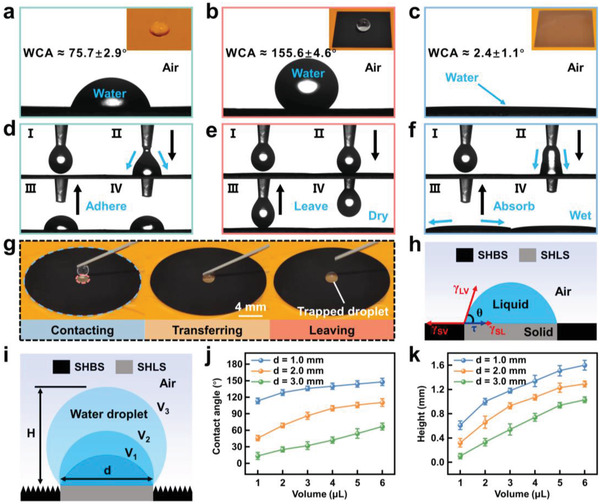
Controllable macroscopic shapes of water droplets on heterogeneous superwettability surface. a–c) Water droplets placed on the PI, HRHPP, and LRLPP surfaces. d–f) Dynamic wetting behavior of a water droplet (≈4 µL) contacting and leaving on the PI, HRHPP, and LRLPP surfaces. g) Typical optical photographs showing the transfer process of a water droplet (≈4 µL) from the microinjector to the SHLS. h) Schematic and mechanical analysis of a liquid droplet on the heterogeneous superwettability surface. i) Schematic of water droplets with different volumes on the SHLS surrounded by SHBS. j) Contact angle and k) height on the SHLS of different diameters as functions of trapped droplet volume.

Then, detailed WCA measurements of the PI, HRHPP, and LRLPP surfaces were performed. The pristine PI film surface is hydrophilic with a WCA of ≈75.7° (**Figure** **3**a). Meanwhile, the HRHPP surface displays superhydrophobicity with a WCA of ≈155.6° (Figure 3b). In addition, the HRHPP surface shows strong water‐repellency to different volumes of water droplets, a small sliding angle (≈2°), and excellent self‐cleaning effects (Figures S9–S11, Supporting Information). Because of the stable surface structures, the WCAs of the HRHPP surface were nearly unchanged when it was placed in the air and at room temperature for a month (Figure S12, Supporting Information). As shown in Figure 3c, the HRHPP surface is superhydrophilic with a WCA of ≈2.4°. Similarly, dynamic wetting behaviors of water droplets on the PI, HRHPP, and LRLPP surfaces were consistent with their WCA values. When a water droplet touched the PI surface, it was transferred from the microsyringe to the PI surface due to water adhesion (Figure 3d). When the water droplet suspending on the microsyringe contacted the HRHPP surface, strong water repulsive from the HRHPP surface caused droplet deformation, but the water droplet was complete and left the HRHPP surface without any loss (Figure 3e). By comparison, when a water droplet contacted the LRLPP surface, the LRLPP surface quickly absorbed the water droplet and was wetted because of the strong water‐absorbing property (Figure 3f). Moreover, a series of sequential optical photos, which were captured using a high‐speed camera, is shown in Figure S13, Supporting Information, to represent water droplet impact behaviors on the PI, HRHPP, and LRLPP surfaces, respectively. When increasing the HRHPP and LRLPP surfaces from 30 to 70 °C or bending them for 50 cycles, their wetting performances had no obvious change, revealing excellent thermal stability and bending resistance (Figure S14, Supporting Information). Detailed wettability change mechanisms of HRHPP and LRLPP surfaces are explained in Figure S15 and Note S1, Supporting Information.^[^ ^48^, ^49^, ^50^, ^51^ ^]^

Based on water repulsion from the SHBS and water adhesion from the SHLS, we introduced a heterogeneous superwettability surface, which is comprised of annular SHBS and circular SHLS, to capture water droplets and form a bulge‐like structure. Figure 3g gives a series of optical photographs of the water droplet transfer process. Because the adhesion ability of superhydrophilicity to water droplets was higher than that of the microinjector, the water droplet was transferred from the microinjector to the SHLS and eventually was trapped on the SHLS. Besides, if a liquid droplet is trapped on the SHLS, the contact angle of the liquid droplet is inconformity with the classical Young equation, because the contribution from line tension to contact angle is incapable to be ignored (Figure 3h). Considering the effect of the line tension, the modified Equation (1) can be expressed as follows^[^ ^52^, ^53^ ^]^

(1) $$\cos\theta =\frac{\gamma_{\mathrm{SV}}-\gamma_{\mathrm{SL}}}{\gamma_{\mathrm{LV}}}-\frac{\tau }{r\gamma_{\mathrm{LV}}}$$

where *θ* is the actual contact angle, *τ* is the line tension, and *r* is the radius of the liquid droplet on the surface. *γ*_SV_, *γ*_SL_, and *γ*_LV_ are the surface tensions of the solid–vapor, solid–liquid, and liquid–vapor interfaces, respectively. The volume of the water droplet determines the line tension, which further affects the contact angle. As illustrated in Figure 3i, with the increase of water droplet volume, the triple‐phase line of the water droplet remains pinned at the boundary between the SHLS and the surrounding SHBS. Even for the huge volume of the water droplet, the water droplet could still be fixed well on the SHLS. Meantime, different volumes of water droplets display different contact angles and heights. Furthermore, we measured the contact angles and heights of water droplets with different volumes on the circular SHLS of different diameters (*d*) (Figure 3j,k). For example, for the SHLS with a diameter of 2 mm, by increasing the water droplet volume from 1 to 6 µL, the contact angle and height can be tuned from 45.7° to 110.3° (angle) and from 0.32 to 1.29 mm (height) (Figure S16, Supporting Information). The experimental results manifest that controlling contact angles and heights of water droplets on the heterogeneous superwettability surface is feasible.

**Figure 4:**
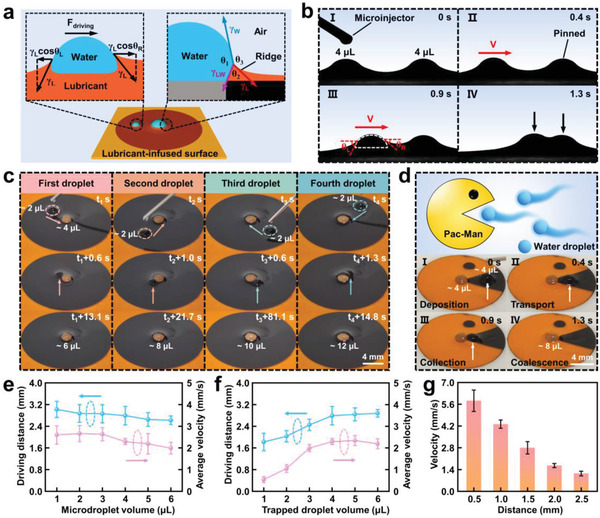
The capacity of directional water self‐transport by capillary force. a) Schematic illustration of the Neumann triangle with the surface tensions (*γ*_L_, *γ*_W_, and *γ*_LW_) and the included angles (*θ*_1_, *θ*_2_, and *θ*_3_) at the triple‐phase (lubricant–water–air) contact line and qualitative mechanism of directional water transport on the LIHSS. b) Optical images showing a typical process of directional water self‐transport on the LIHSS. c) Transporting and collecting microdroplets from different directions. d) Patterned LIHSS to imitate the popular Pac‐Man game. Maximum self‐driving distance and average velocity of the microdroplet as functions of e) microdroplet volume and f) trapped droplet volume. g) Velocity of the microdroplet as a function of the distance between the microdroplet and the trapped droplet. The volumes of the trapped droplet and microdroplet are ≈4 µL.

The femtosecond laser can directly prepare 3D porous micro/nanostructures on various substrates, which is beneficial for the efficient fabrication of slippery surfaces.^[^ ^54^, ^55^, ^56^, ^57^, ^58^, ^59^, ^60^, ^61^ ^]^ The femtosecond laser‐treated PI film surfaces show high chemical affinity to various lubricants including silicone oil, white oil, and 1‐decanol (Figure S17, Supporting Information). Consequently, the lubricant was infused into the micropores of the heterogeneous superwettability surface and formed a thin lubricant film, which bestowed unique slippery property on the substrate. It was manifested that a water droplet could easily slide off on the silicone oil‐infused SHBS with a small sliding angle of ≈2° (Figure S18, Supporting Information). Importantly, as illustrated in **Figure** **4**a, the trapped water droplet on the LIHSS was surrounded by a lubricant wetting ridge owing to the surface tension. At the triple‐phase contact line of lubricant–water–air, the three surface tensions (lubricant–air, water–air, and lubricant–water) mutually form three included angles, which are satisfied with the Neumann triangle rule and Equations (2)–(4)^[^ ^62^, ^63^ ^]^

(2) $$\cos\theta_{1}=\frac{\gamma_{L}^{2}-\gamma_{W}^{2}-\gamma_{\mathrm{LW}}^{2}}{2\gamma_{W}\gamma_{\mathrm{LW}}}\;$$

(3) $$\cos\theta_{2}=\frac{\gamma_{W}^{2}-\gamma_{L}^{2}-\gamma_{\mathrm{LW}}^{2}}{2\gamma_{L}\gamma_{\mathrm{LW}}}\;\;$$

(4) $$\cos\theta_{3}=\frac{\gamma_{\mathrm{LW}}^{2}-\gamma_{W}^{2}-\gamma_{L}^{2}}{2\gamma_{W}\gamma_{L}}\;\;$$

where *γ*_L_, *γ*_W_, and *γ*_LW_ are the surface tensions of the lubricant, water, and lubricant–water interfaces, respectively. *θ*_1_, *θ*_2_, and *θ*_3_ are the Neumann angles between the various surface tensions. The Neumann angles are related to the physical properties of lubricant involving surface tension and solubility.^[^ ^64^ ^]^ Thus, the water droplets on the slippery surfaces demonstrated different macroscopic shapes when changing the lubricants (Figure S19, Supporting Information). Therefore, the LIHSS is an ideal platform for discussing the interaction between water droplets and various kinds of lubricants.

When a microdroplet was placed on the LIHSS, the wetting ridges of the microdroplet and the trapped droplet occurred to overlap and were lifted up, generating a capillary driving force for transporting the microdroplet to the trapped droplet (Figure 4a). The capillary driving force (*F*_driving_) can be expressed by the following Equation (5)^[^ ^34^, ^35^ ^]^

(5) $$\;F_{\mathrm{driving}}=\gamma_{L}\;R\left(\cos\theta_{R}-\cos\theta_{L}\right)\;$$

where *R* is the microdroplet size, *θ*_R_ and *θ*_L_ are the horizontal angles of the wetting ridge to the right and left side of the microdroplet, respectively. Taking advantage of the unique slippery property and lubricant wetting ridge‐induced capillary driving force, the LIHSS exhibits excellent performance in directional water self‐transport. From Figure 4b, it was observed that a microdroplet (≈4 µL) could be transported to the trapped droplet (≈4 µL) at a distance of 2.7 mm within 1.3 s (Movie S1, Supporting Information). During the process, the microdroplet was in accelerated movement until it collided with the trapped droplet, while the trapped droplet remained pinned on the SHLS. In addition, the as‐prepared slippery surfaces were still able to transport water droplets when the lubricant was varied from white oil to 1‐decanol, demonstrating the lubricant selection generalization of the LIHSS (Movie S2 and Figure S20, Supporting Information). As for the LIHSS with a large volume trapped droplet, the microdroplet (≈4 µL) could also be driven rapidly to the large volume trapped droplet (Figure S21, Supporting Information). Moreover, we investigated the water droplet dynamic behaviors on the lubricant‐infused superwettability surface (Figure S22, Supporting Information). Results show that the droplets tended to approach and collide on the lubricant‐infused SHBS, whereas the droplets pinned instead of moving on the lubricant‐infused SHLS (Movie S3, Supporting Information). It is worthwhile mentioning that the trapped droplet can be replaced by some other objects, such as a small steel ball, which raises the lubricant wetting ridge and achieves directional water self‐transport (Figure S23, Supporting Information). Notably, our strategy for directional water self‐transport could also realize transporting and collecting of droplets from different directions. As shown in Figure 4c, four water droplets (≈2 µL) were successively placed on the same LIHSS from four different directions: the upper left, the lower left, the lower right, and the upper right. During the process, the four droplets were successfully transported to the central trapped droplet and coalesced (Movie S4, Supporting Information). Apart from the ability for transporting droplets from different directions, continuously driving droplets from the same location could be achieved (Movie S5 and Figure S24, Supporting Information). The renewable fluidic lubricant wetting ridge is conducive to continuous driving droplets capability.

Drawing support from the flexibility of femtosecond laser treatment, we can design and fabricate various patterned slippery surfaces. As can be seen from Figure 4d, a heterogeneous superwettability surface consisting of circular SHLS and a fan‐shaped SHBS was prepared to imitate the popular Pac‐Man game. A water droplet (≈4 µL) was deposited on the lubricant‐infused fan‐shaped SHBS and finally was collected by the central trapped droplet (≈4 µL), reflecting a water droplet‐eating process (Movie S6, Supporting Information). Likewise, the patterned central SHLS could also achieve water self‐transport (Figure S25, Supporting Information).

To explore the capability of directional water self‐transport, we investigated the relationship between the volume of water droplets and self‐transport parameters including maximum self‐driving distance and average velocity. The average velocity of microdroplets was calculated by dividing the maximum self‐driving distance by the time to transport. With the increase of microdroplet volume from 1 to 6 µL, the maximum self‐driving distance and average velocity are slightly lowered when the trapped droplet volume is ≈4 µL (Figure 4e). Within the range, the maximum self‐driving distance can reach ≈3 mm. This result indicates that the maximum self‐driving distance is relatively insensitive to the volume of microdroplets and the driving strategy applies to most droplet sizes. Theoretically, the self‐driving distance is mainly determined by the length of the lubricant wetting ridge, which is given by the capillary length. The capillary length (*λ*) can be expressed by the following Equation (6)^[^ ^35^, ^65^ ^]^:

(6) $$\lambda \;=\sqrt{\frac{\gamma_{L}}{\rho_{L}g}}\;$$

in which *λ* is termed as the capillary length, *ρ*_L_ is the lubricant density, and *g* is the gravitational acceleration. For the silicone oil (≈10 cst), the density *ρ*_L_ ≈ 0.935 g cm^−3^, the surface tension *γ*_L_ ≈ 20.1 mN m^−1^, the gravitational acceleration *g* ≈ 9.8 m s^−2^, and the capillary length *λ* ≈ 1.48 mm. Therefore, the theoretic maximum self‐driving distance is twice that of the capillary length, and this means a maximum self‐driving distance of ≈2.96 mm, which is in agreement with experimental data. Besides, we studied the self‐driving capability of different volumes of trapped droplets. The result suggests that with the increase of trapped droplet volume, the maximum self‐driving distance and average velocity raise first and then level off when the microdroplet volume is ≈4 µL (Figure 4f). In addition, the velocity of the microdroplet increases with the decreasing distance from the microdroplet to the trapped droplet (Figure 4g). This may be caused by the change in the horizontal angles of the wetting ridge on both sides.

**Figure 5:**
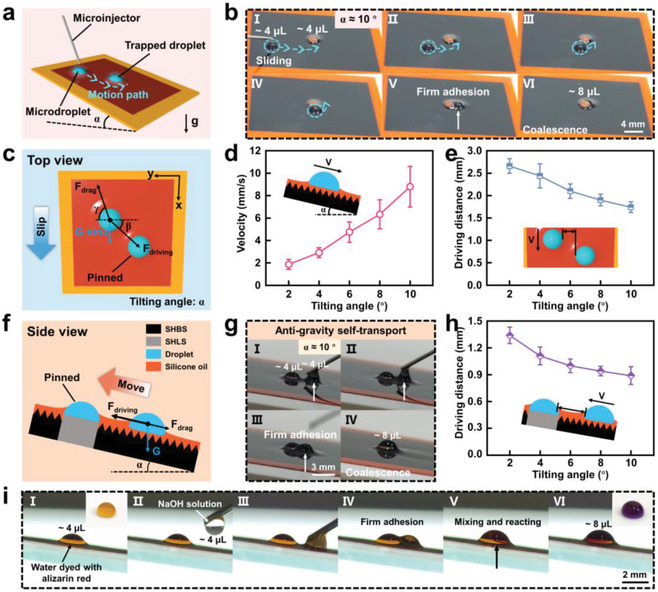
Water self‐transport and applications on the tilted LIHSS. a) Schematic of water self‐transport process on the tilted LIHSS. b) Water self‐transport process on the tilted LIHSS with a tilting angle of ≈10°. c) Force analysis of the microdroplet on the tilted LIHSS. d) Relationship between velocity and tilting angle at the droplet volume of ≈4 µL. e) Maximum self‐driving distance as a function of the tilting angle. The volumes of the trapped droplet and microdroplet are ≈4 µL. f) Force analysis of the microdroplet during the anti‐gravity water self‐transport process. g) Anti‐gravity water self‐transport process on the tilted LIHSS with a tilting angle of ≈10°. h) Maximum anti‐gravity self‐driving distance as a function of the tilting angle. The volumes of the trapped droplet and microdroplet are ≈4 µL. i) Optical images of a simple chemical microreaction process based on anti‐gravity droplet self‐transport and droplet positioning coalescence. The insets show the different colors of the corresponding droplets on the polytetrafluoroethylene (PTFE) film surface.

To further demonstrate the potential applications of the LIHSS, we extended water self‐transport to the tilted surface and carried out two experiments involving anti‐gravity transport and chemical microreaction. **Figure** **5**a illustrates a schematic diagram of water self‐transport on the tilted LIHSS. The microdroplet slides off under the effect of gravity acceleration and is attracted by the trapped droplet through capillary driving force. Owing to strong water adhesion from SHLS, the trapped droplet is fixed on the tilted surface. Figure 5b manifests the water self‐transport process on the tilted LIHSS when the tilting angle is ≈10°. It was found that the microdroplet (≈4 µL) slid, moved in a curved path toward the direction of the trapped droplet (≈4 µL), and finally coalesced with it (Movie S7, Supporting Information). Then, the mechanism is analyzed by constructing a mechanical model. During curvilinear motion, the gravity along the tilted direction and capillary force, as the driving force, transported the microdroplet to the trapped droplet (Figure 5c)

(7) $$F_{\mathrm{driving}}\cos\beta >F_{\mathrm{drag}}\cos\gamma$$

(8) $$\;F_{\mathrm{driving}}\sin\beta +G\sin\alpha >F_{\mathrm{drag}}\sin\gamma \;$$

where *F*_drag_ is the viscous drag force, which is always opposite to the velocity. *G* is the gravity. *α* is the tilting angle, *β* is the angle between *F*_driving_ and *y* direction, and *γ* is the angle between *F*_drag_ and *y* direction. The influence of the tilting angle (*α*) on the microdroplet velocity (*V*) and maximum self‐driving distance (*D*) was investigated. It was found that the greater the tilting angle, the faster the velocity of the microdroplet (Figure 5d). Accordingly, as the tilting angle increases from 2° to 10°, the maximum self‐driving distance decreases from 2.66 to 1.74 mm (Figure 5e). The main reason is that when the velocity of the microdroplet becomes faster, the time required to pass the surround of the trapped droplet is shorter, contributing to the decrease of maximum self‐driving distance.

Multifunctional transport and coalescence of water droplets on the tilted LIHSS can be harnessed for anti‐gravity water self‐transport and chemical microreaction. As described in Figure 5f, when a microdroplet is in a location that is close to the horizontal plane, the capillary force can simultaneously overcome the resistance of gravity along the tilted direction and viscous drag force

(9) $$F_{\mathrm{driving}}>F_{\mathrm{drag}}+G\sin\alpha \;$$

when the capillary force induced by the lubricant wetting ridge is larger than the sum of the gravity along the tilted direction and viscous drag force, the microdroplet begins to move toward the direction of the trapped droplet. As shown in Figure 5g, it could be seen that on the tilted LIHSS at a tilting angle of ≈10°, the microdroplet (≈4 µL) was spontaneously transported to the trapped droplet (≈4 µL) and finally coalesced together to form a larger droplet (≈8 µL) (Movie S8, Supporting Information). We also developed the influence of tilting angle on the performance of anti‐gravity water self‐transport. The result shows that with a rising tilting angle, a gradual decrease of maximum anti‐gravity self‐driving distance is observed, which is in accordance with the increase of the microdroplet gravity along the tilted direction (Figure 5h).

In addition, we performed an experiment on liquid–liquid chemical microreaction using water droplets (Figure 5i). First, a water droplet (≈4 µL) dyed with alizarin red was deposited on the central SHLS, and the dyed water manifested with orange. Then, a droplet of NaOH solution (≈4 µL) was added around the dyed water droplet. Under the effect of capillary force, the NaOH solution droplet was actuated to move toward the direction of the dyed water droplet and coalesce with the dyed water droplet (Movie S9, Supporting Information). Since the merged droplet was alkaline, the alizarin red molecules changed their existence form, which transformed the color of the droplet from orange to purple. In short, we demonstrated that water reactant microdroplets can transport, coalesce, and react on the tilted LIHSS, which can be used as a platform for chemical microreaction.

## 3 Conclusion

In summary, inspired by nature, a LIHSS was designed on the PI film and fabricated by a powerful method of femtosecond laser direct writing and lubricant infusion, which successfully realized directional water self‐transport. Because of the strong water adhesion of the SHLS, water droplets with different volumes could be trapped on the heterogeneous superwettability surface and manifested with different macroscopic shapes. After the deposition of excess lubricant, microdroplets on the LIHSS could be transported to the trapped droplet by a wetting ridge‐induced capillary driving force. Also, the maximum self‐driving distance could reach ≈3 mm. Moreover, the trapped droplet was capable of continuously attracting microdroplets in any direction. Finally, we demonstrated that water self‐transport, anti‐gravity water droplet pumping, and chemical microreaction were achieved on the tilted LIHSS, which manifested its enormous potential in microdroplet manipulation. From a broader perspective, we believe that our research can provide new insights into the development of multifunctional slippery surfaces and directional water self‐transport.

## 4 Experimental Section

### Materials

In this experiment, PI film with a thickness of 0.1 mm was purchased from Zhongshan Chenxi Electronics Co. Ltd (Guangdong, China). Silicone oil (PMX‐200, ≈10 cst) was supplied by Aladdin Industrial Co. Ltd (Guangdong, China). 7# white oil was obtained from Linyi Yiqun Chemical Industry Co. Ltd (Shandong, China). 1‐decanol was achieved from Shandong Yousuo Chemical Science Co. Ltd (Shandong, China). Alizarin red was commercially available from Tianjin Dengfeng Chemical Reagent Factory (Tianjin, China).

### Femtosecond Laser Fabrication

The laser beam (pulse width of 350 fs; central wavelength of 1035 nm) was generated by a commercial femtosecond fiber laser system (HR‐Femto‐IR‐50‐40B, Huaray, China). Through a two‐mirror galvanometric scanner system (basiCube 10, Scanlab, Germany) equipped with an F‐Theta lens (focused length of 125 mm), the laser beam was guided onto the sample surface and scanned along the *x*–*y* coordinate direction. The scanning path was designed by EzCad software. The laser scanning speed and scanning interval were set at 50 mm s^−1^ and 20 µm, respectively. The laser repetition rate and power were respectively set at 200 kHz and 700 mW to obtain SHBS. In contrast, the laser repetition rate and power were respectively set at 5 kHz and 40 mW to obtain SHLS. All the experiments were carried out in the air and at room temperature. In the experiment, silicone oil (≈10 cst) was selected as the main lubricant and the diameter of the circular SHLS was 2 mm.

### Instrument and Characterization

The morphologies and structures of the samples were characterized by a field emission SEM (MIRA3 LMU, TESCAN, Czech Republic). The chemical compositions and elemental maps were determined using energy‐dispersive X‐ray spectroscopy (EDS, TESCAN, Czech Republic). The 3D surface morphologies and cross‐sectional profiles were taken by LCM (Axio LSM700, Zeiss, Germany). A UV–vis spectrophotometer (UV‐2600, Japan) with an integrating sphere was used to investigate the transmissivity and reflectivity of samples in the range of 220–1400 nm. The contact angles of different liquid droplets were measured with a contact angle measurement system (Biolin Scientific, Finland). The average contact angles and standard deviations were calculated by measuring five droplets at different locations on the same sample surface.

## Conflict of Interest

The authors declare no conflict of interest.

## Supporting information

Supporting Information

Supplemental Movie 1

Supplemental Movie 2

Supplemental Movie 3

Supplemental Movie 4

Supplemental Movie 5

Supplemental Movie 6

Supplemental Movie 7

Supplemental Movie 8

Supplemental Movie 9

## Data Availability

The data that support the findings of this study are available from the corresponding author upon reasonable request.
