# Supplementary material for: Wetting Ridge‐Guided Directional Water Self‐Transport
Source: Adv Sci (Weinh). 2022 Oct 17;9(34):2204891. doi: 10.1002/advs.202204891 (PMC9731720; doi:10.1002/advs.202204891)
Supplement: Supplementary file 1 — Supporting Information [file ADVS-9-2204891-s002.pdf]

Supporting Information

Wetting Ridge-Guided Directional Water Self-Transport

Lingxiao Wang [a], *Kai Yin* [a,b,]*, *Qinwen Deng* [a], *Qiaoqiao Huang* [a], *Jun He* [a], *Ji-An Duan* [b]

[a] *Hunan Key Laboratory of Nanophotonics and Devices, School of Physics and Electronics, Central South University, Changsha, 410083, China.*

[b] *The State Key Laboratory of High Performance and Complex Manufacturing, College of Mechanical and Electrical Engineering, Central South University, Changsha 410083, China.*

**Corresponding* author: *kayin@csu.edu.cn (K. Yin)*

This file contains Supplementary Figure S1-24, Note S1 and Movie S1-9.

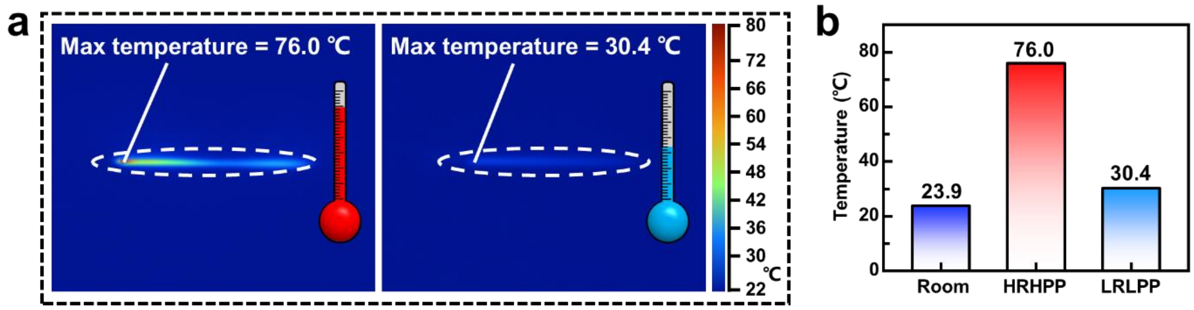

Figure S1. a) IR thermal images showing the maximum temperatures during the fabrication processes of HRHPP and LRLPP surfaces. b) Comparison of room temperature, HRHPP, and LRLPP, indicating divergent femtosecond laser thermal accumulation effects.

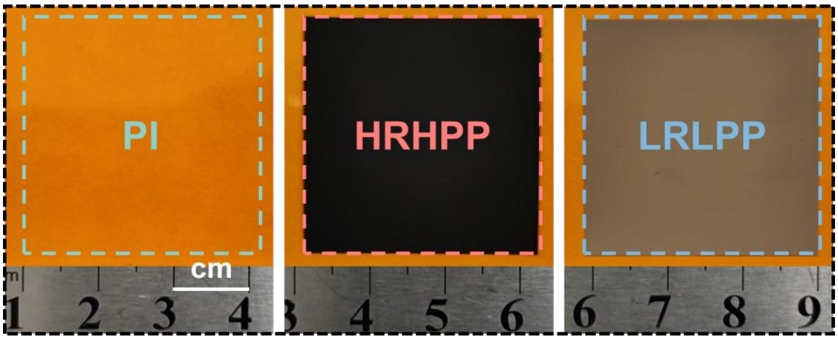

Figure S2. Optical photos of PI, HRHPP, and LRLPP surfaces. The three surfaces appeared orange, black, and grey, respectively.

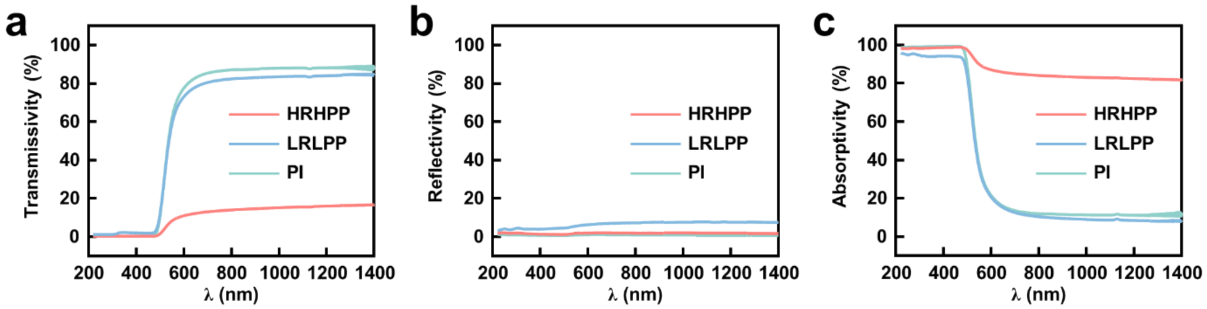

Figure S3. a) Transmissivity, b) reflectivity, and c) absorptivity of the PI, HRHPP, and LRLPP surfaces in the range of 220–1400 nm.

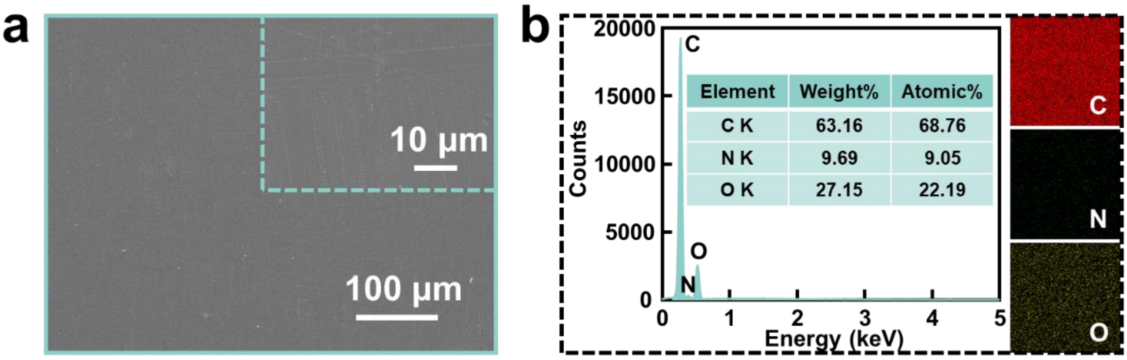

Figure S4. a) SEM images of the pristine PI surface. b) Elemental chemical composition and mapping images of C, N and O of the PI surface.

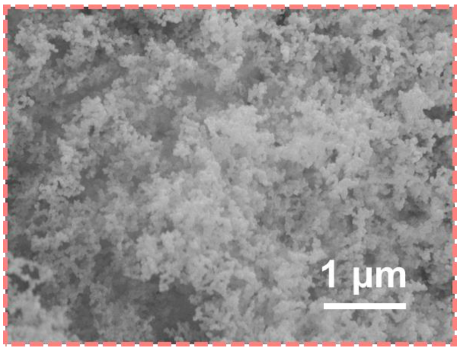

Figure S5. High-magnification SEM image of protrusions on the HRHPP surface. The SEM image showed that the protrusions are covered with nanoparticles.

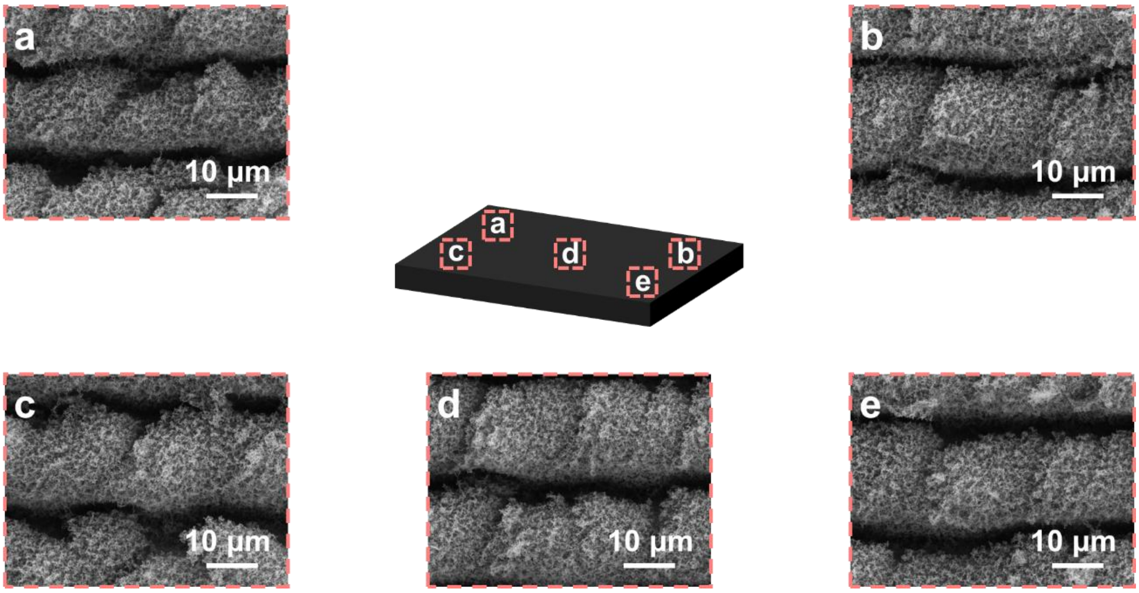

Figure S6. SEM images of HRHPP surface at five different locations.

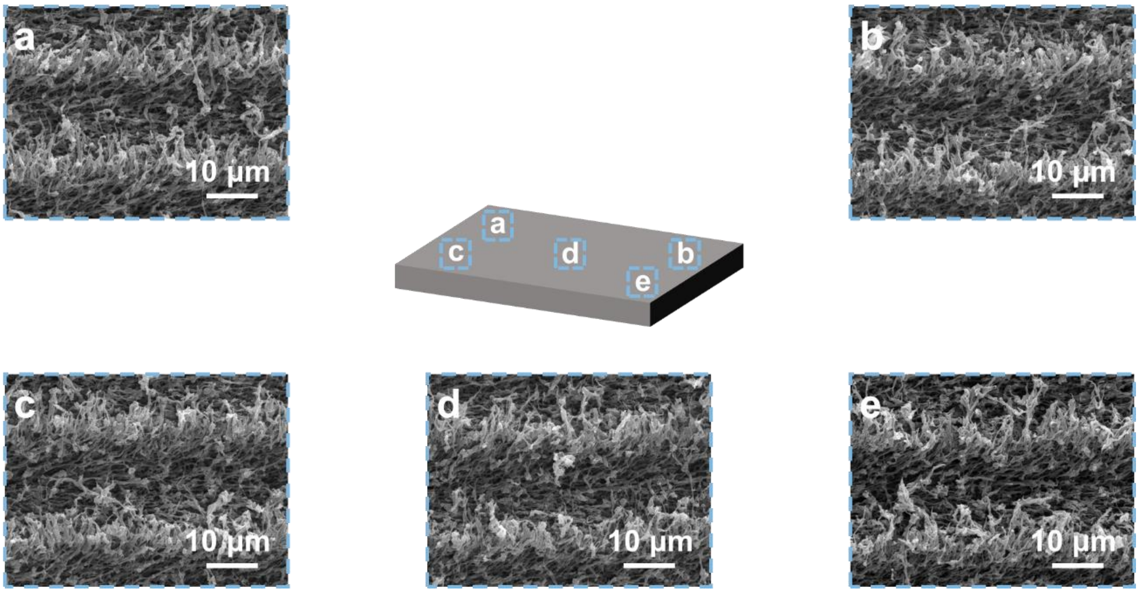

Figure S7. SEM images of LRLPP surface at five different locations.

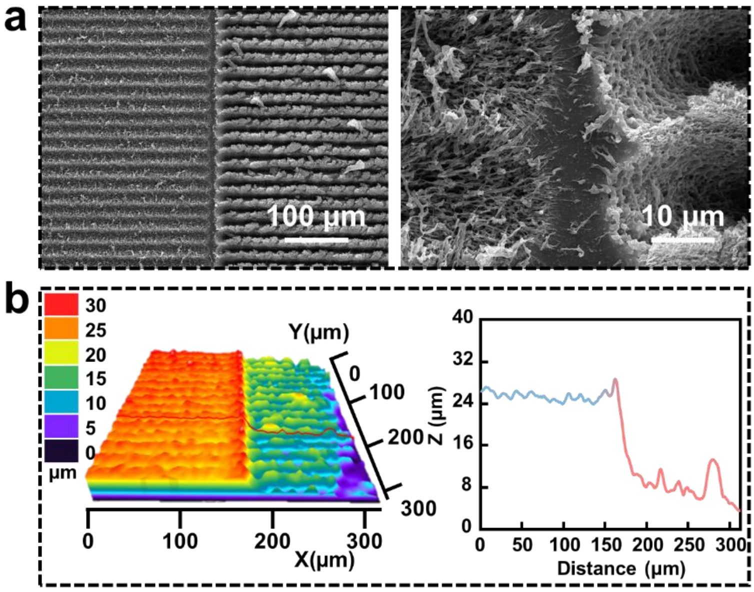

Figure S8. (a) SEM images, b) 3D confocal image and cross-sectional profile of the boundary line between superhydrophilic and superhydrophobic surfaces.

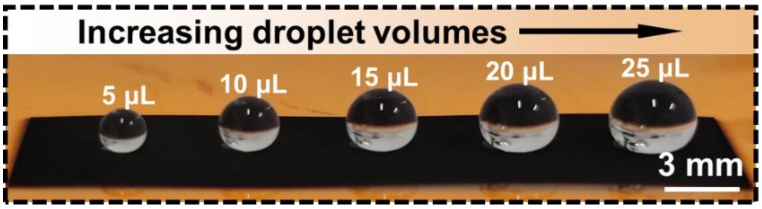

Figure S9. Optical photo for water droplets with different volumes (~5 μL, ~10 μL, ~15 μL, ~20 μL, and ~25 μL) on the HRHPP surface, showing its strong water-repellency.

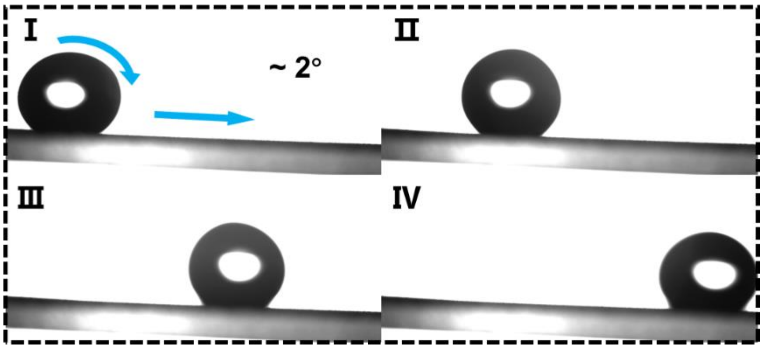

Figure S10. Time-resolved optical images of a water droplet sliding (~2 ◦) on the HRHPP surface.

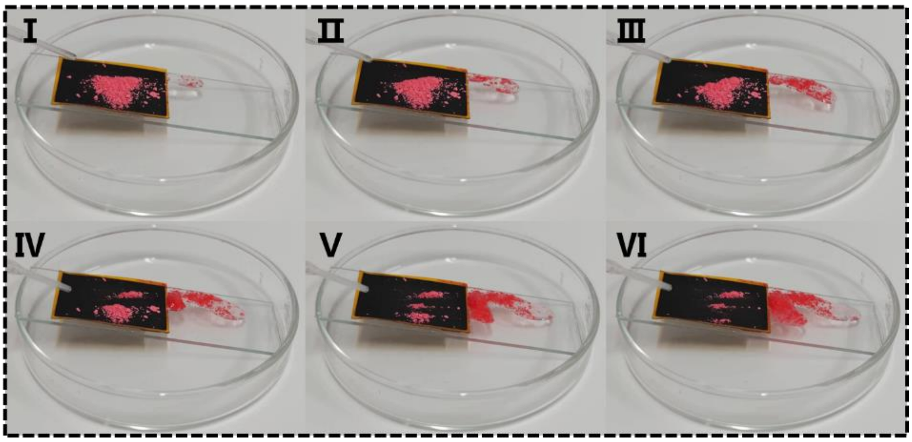

Figure S11. Self-cleaning demonstration of the HRHPP surface. Pink chalk powder was sprinkled on the tilted HRHPP surface to simulate common dust. Because the water droplets slid off on the HRHPP surface, the chalk powder was easily collected and removed by the water droplets.

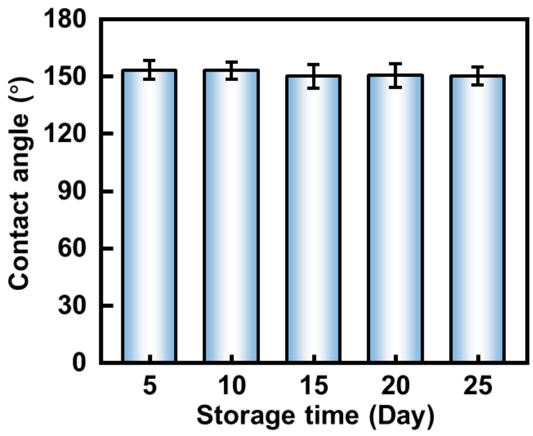

Figure S12. Water contact angles of the HRHPP surface placed in air and room temperature for a month. The water contact angles of HRHPP surface were measured per five days under a condition of temperature ~25 °C and humidity ~40 %.

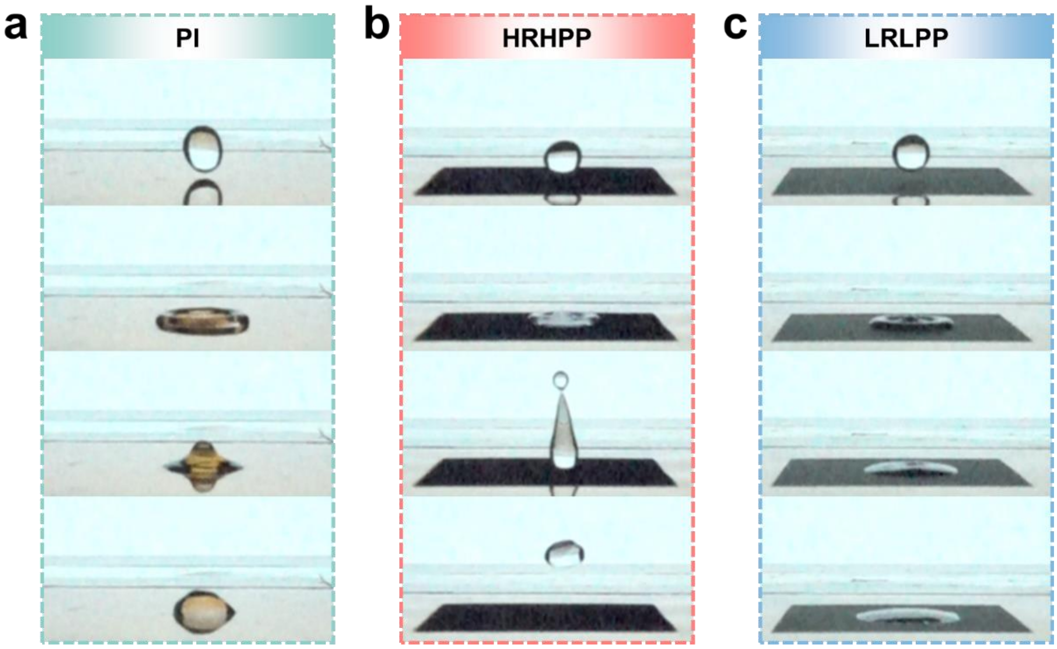

Figure S13. Sequential optical photos of water droplets impact on the a) PI, b) HRHPP, and c) LRLPP surfaces.

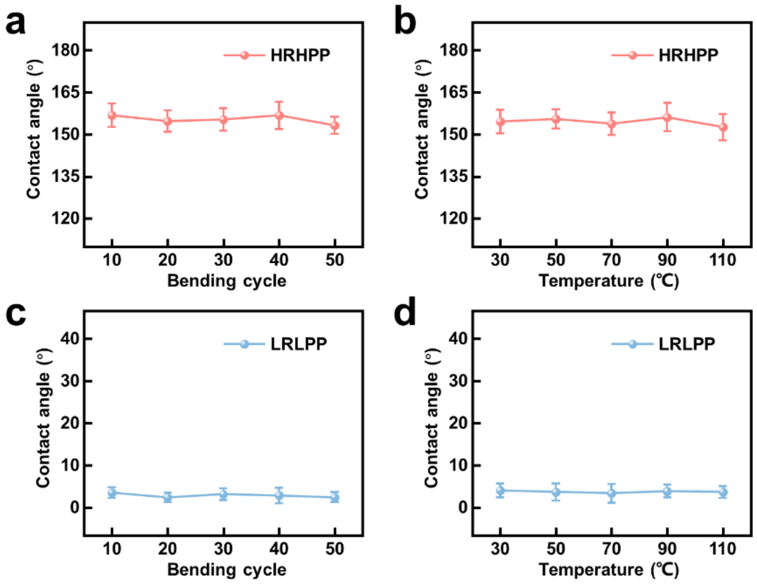

Figure S14. Water contact angles of the a), b) HRHPP and c), d) LRLPP surfaces in the resisting bending and thermal stability tests.

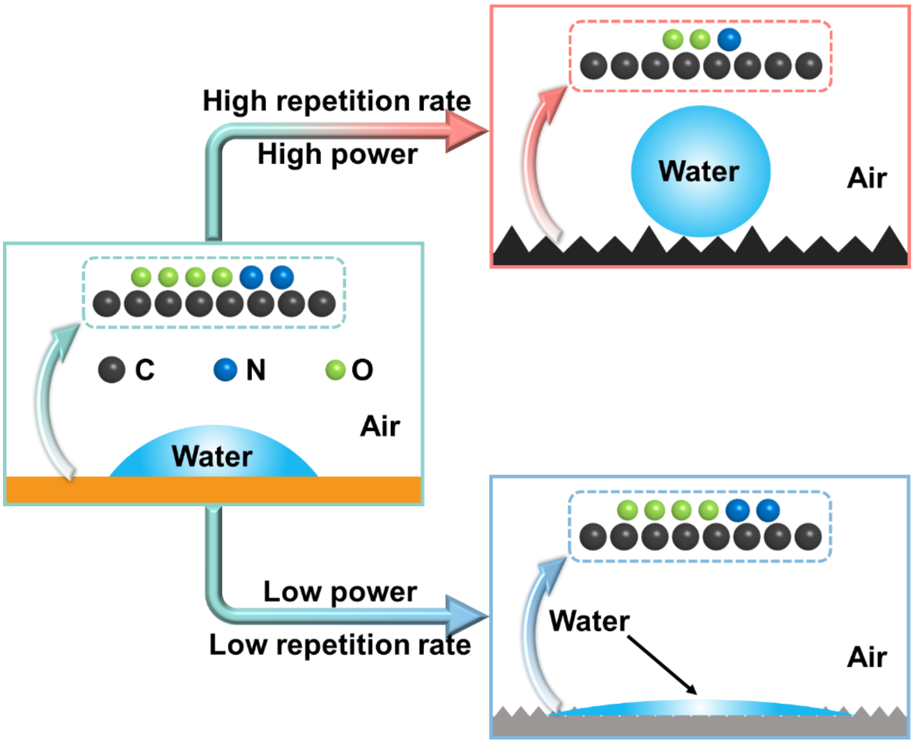

Figure S15. Mechanism illustration of LRLLP and HRHLP surfaces superwettability. The

corresponding wettability change mechanisms are explained in Note S1.

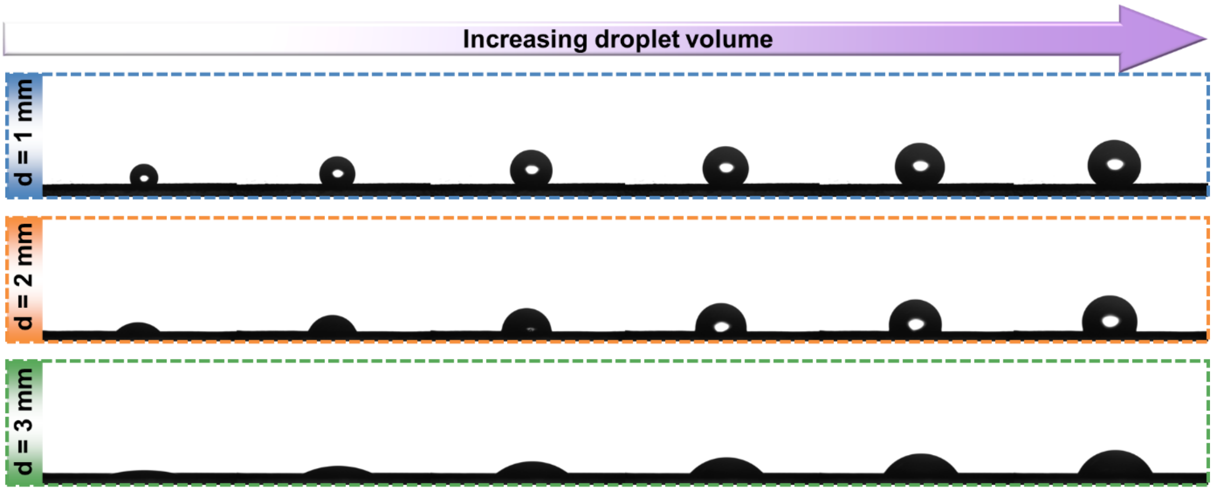

Figure S16. Optical images of the water droplets placed on the heterogeneous superwettability surface with an increasing volume from 1 μL to 6 μL. The diameters of the circular superhydrophilic surfaces are 1 mm, 2 mm, and 3 mm, respectively.

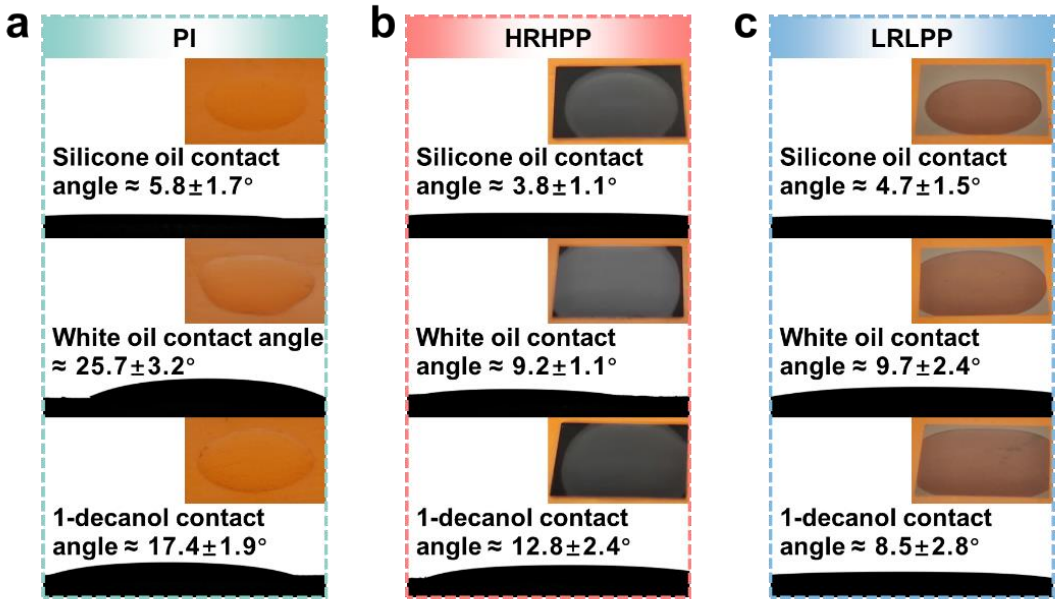

Figure S17. Static lubricant (silicone oil, white oil, and 1-decanol) contact angles of the a) PI, b) HRHPP, and c) LRLPP surfaces, demonstrating high chemical affinity of the sample

surfaces after femtosecond laser treatment.

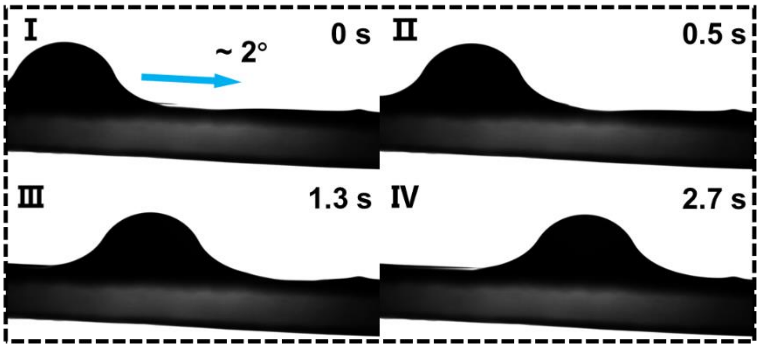

Figure S18. A water droplet sliding off on the silicone oil-infused superhydrophobic surface. The sliding angle is ~2°.

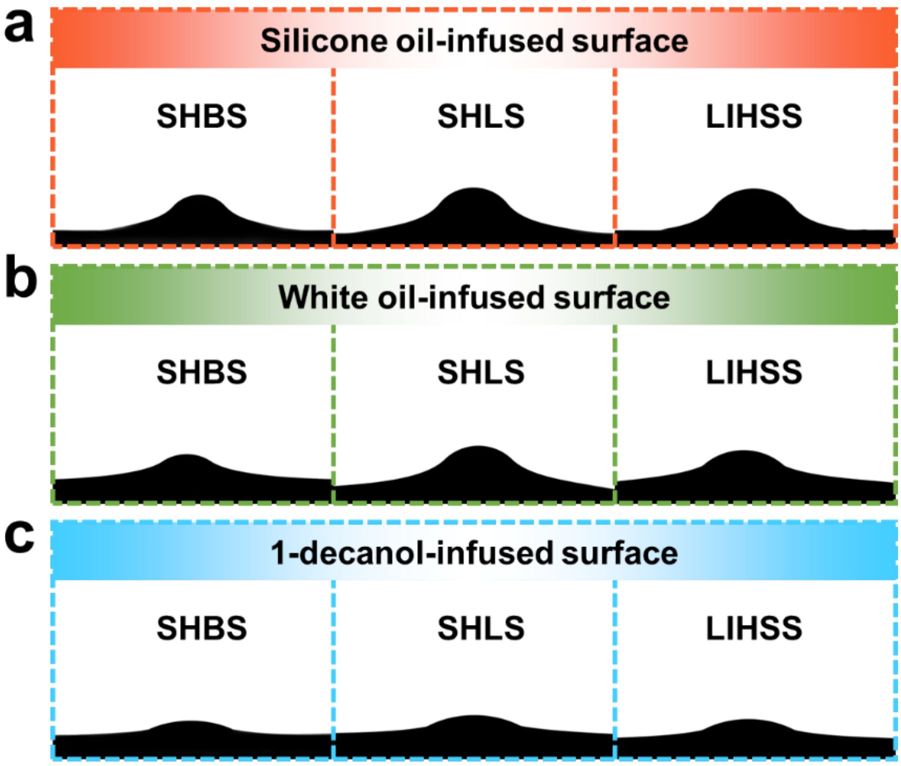

Figure S19. Optical images of the water droplets deposited on the lubricant-infused slippery surface, including superhydrophobic, superhydrophilic, and heterogeneous superwettability

surfaces. The lubricant involves a) silicone oil, b) white oil, and c) 1-decanonl. The water droplet volume is ~4 μL. Lubricant wetting ridge can be clearly seen from the optical images.

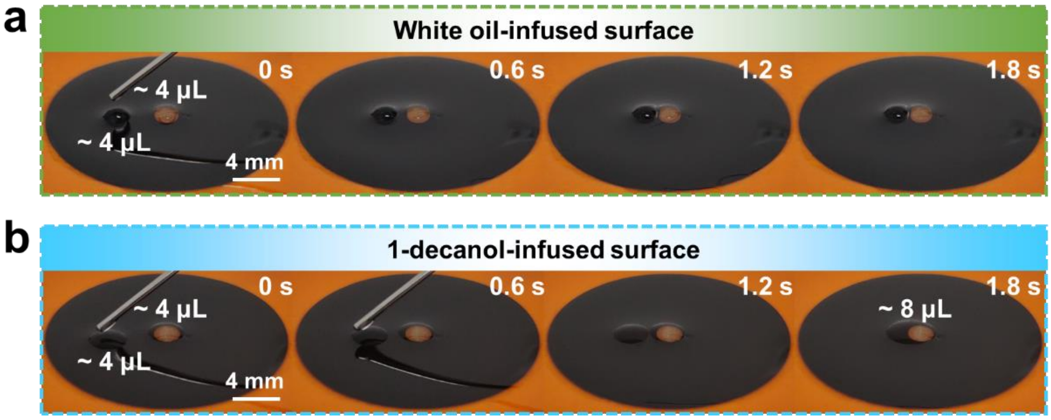

Figure S20. Optical photos of directional microdroplet transport on the lubricant-infused heterogeneous superwettability surface. a) White oil and b) 1-decanol are selected as the lubricant to demonstrate lubricant selection generalization.

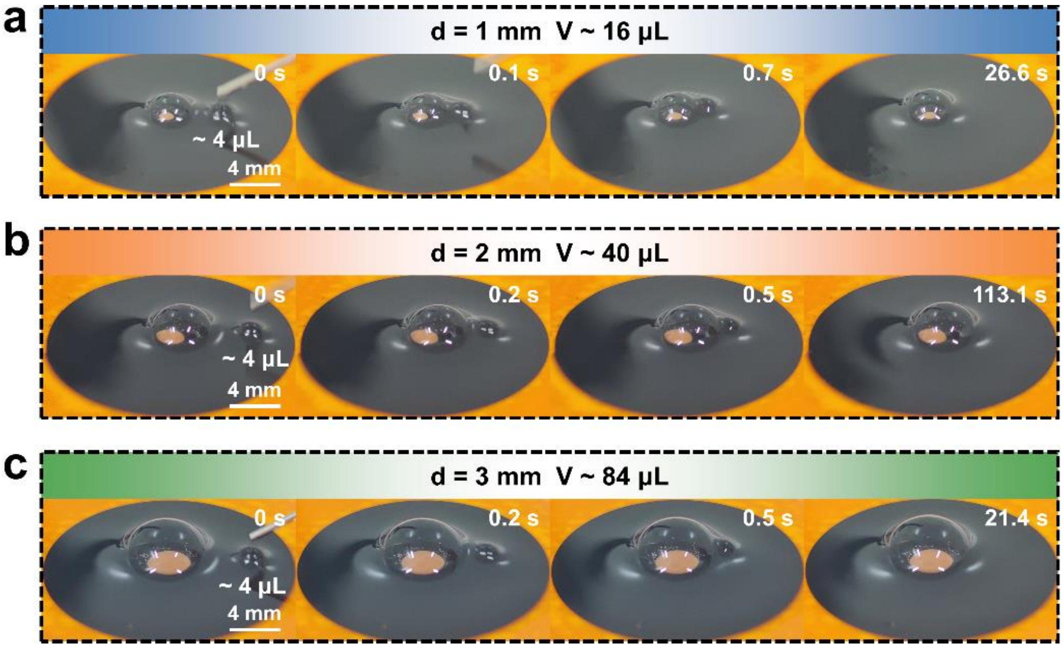

Figure S21. Directional self-transport of water droplets on the lubricant-infused heterogeneous superwettability surface with a large volume trapped droplet. The diameters of

superhydrophilic are 1 mm, 2 mm, and 3 mm. Correspondingly, the volumes of trapped droplets are ~16 μL, ~40 μL, and 84 μL.

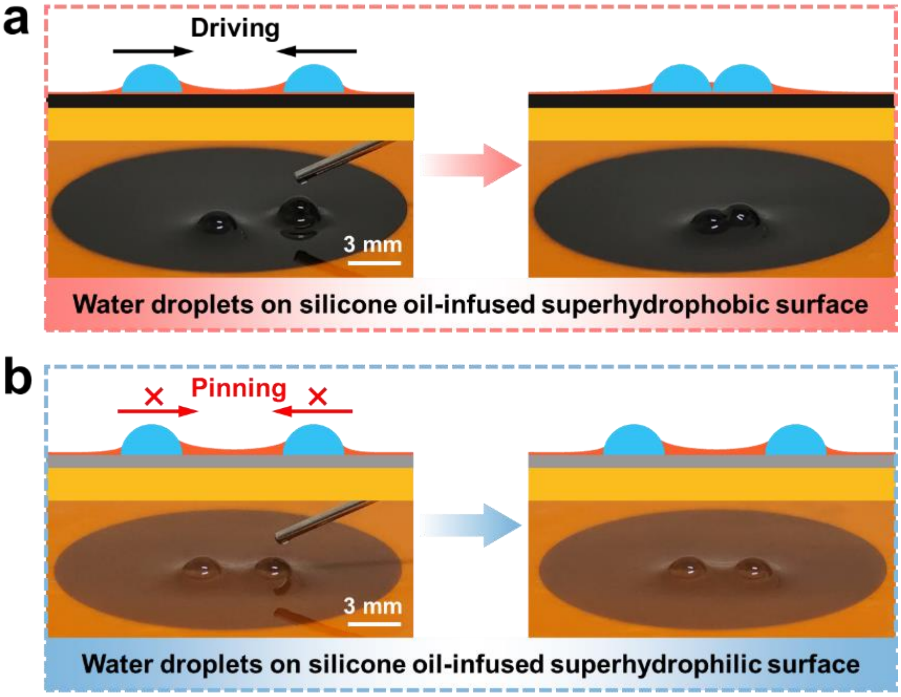

Figure S22. Water droplet dynamic behaviors on the silicone oil-infused superwettability surface. a) Water droplets tended to approach on the lubricant-infused superhydrophobic surface owing to ultralow adhesion force between water and lubricant. b) Water droplets pinned on the lubricant-infused superhydrophilic surface due to adhesion force between water and superhydrophilic surface. The water droplet volume is ~4 μL.

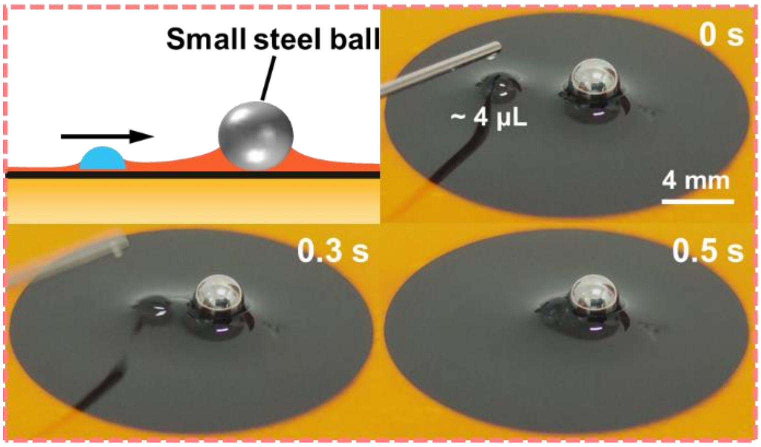

Figure S23. Driving microdroplet by lubricant wetting ridge immobilized by a small steel ball on the silicone oil-infused superhydrophobic surface. Result indicated that directional water

self-transport could be achieved as long as the lubricant wetting ridge existed on the slippery surface.

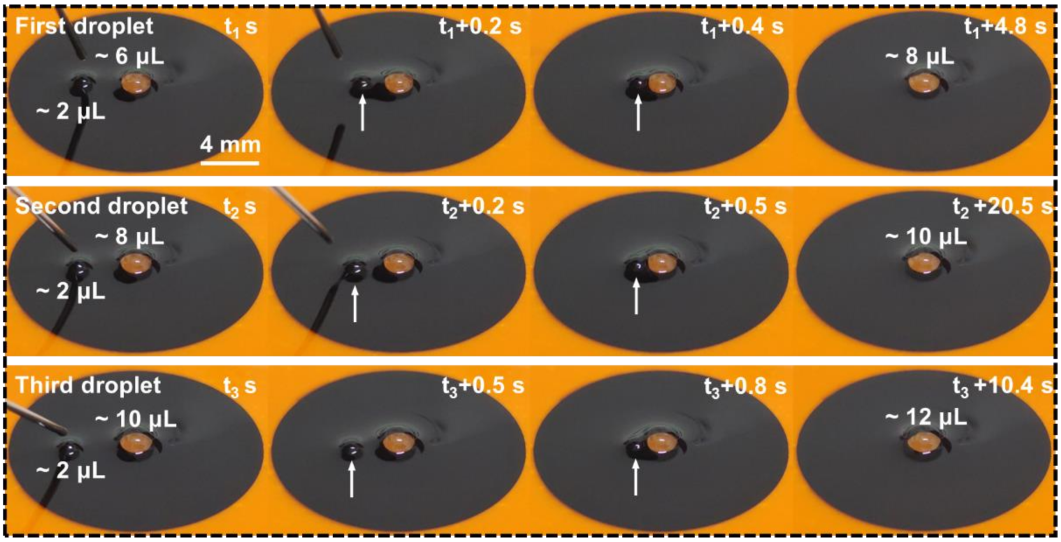

Figure S24. Continuous driving capability of the silicone oil-infused heterogeneous superwettability surface. Because lubricant wetting ridge regenerated after each droplet transport, three microdroplets were continuously transported to the trapped droplet and coalesced together.

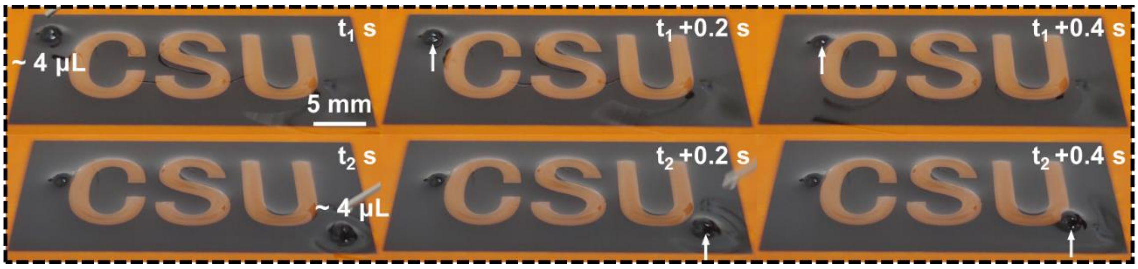

Figure S25. Water droplets self-transport on the patterned silicone oil-infused heterogeneous superwettability surface. The optical photos show that the two microdroplets were transported to the trapped droplet on the patterned superhydrophilic surface.

Note S1. Detailed illustration for wettability change mechanisms of HRHPP and LRLPP surfaces.

In general, intrinsic contact angle is decided by chemical compositions of the sample surface, whereas apparent contact angle is determined by surface structures. Water droplets placed on rough surfaces may exist in different wetting states and exhibit different static wetting behaviors.

In our experiment, the pristine polyimide (PI) surface is hydrophilic with a water contact angle (WCA) of ~75.7 °. After high repetition rate and high power femtosecond laser treatment, the sample surface is rough and has a lower content of N and O element. Based on the experiment data, a water droplet on the HRHPP surface is unable to wet the sample surface, which is consistent with Cassie-Baxter state. In this situation, Cassie-Baxter state and Equation (S1) are used to elucidate the superhydrophobicity:

$$\cos\theta_{CB} = f\cos\theta_Y^* + f - 1 \qquad \text{(S1)}$$

where θ_{CB} is the apparent WCA, f is the fraction of the HRHPP surface in contact with water, and θ^*_Y is the intrinsic WCA of the flat surface. Owing to the decrease content of N and O element, the interaction force between water and HRHPP surface decreases, allowing for an increase of intrinsic WCA. The flat surface with the same chemical composition as the HRHPP surface even might be hydrophobic. In addition, the HRHPP surface displays porous group floc-like microstructures with a higher surface roughness, which is beneficial to the existence of air pockets between water and microstructures. This feature leads to a lower f and an increase of apparent WCA (θ^*_Y), further enhanced the hydrophobicity. Finally, the HRHPP surface is superhydrophobic with a WCA of ~155.6 °. In brief, the combined effect of surface roughness and chemical composition bestows superhydrophobic property on the HRHPP surface.

By comparison, the PI surface treated with low repetition rate and low power femtosecond laser exhibits porous filiform microstructures and superhydrophilic property. A water droplet

on the LRLPP surface can completely wet the surface, which is explained by Wenzel state. To illuminate the superhydrophilicity, Wenzel state and Equation (S2) are employed:

$$\cos\theta_W = r\cos\theta_Y \quad \text{(S2)}$$

where θ_W is the apparent WCA, r is the roughness ratio defined as the ratio of the actual contact area to projected area, and θ_Y is the intrinsic WCA of the smooth surface. Because the contents of C, N, and O elements of LRLPP surface have barely changed compared with the elements of the pristine PI surface, the intrinsic WCA (θ_Y) is of the same as the WCA of pristine PI surface in numeral. From the above Equation S2, the porous filiform microstructures on the LRLPP surface produce a surface roughness and enhance the hydrophilicity. As a result, the LRLPP surface exhibits superhydrophilicity (~2.4 °). Namely, the wettability amplifying function of porous filiform microstructures endow the LRLPP surface with superhydrophilicity.

Movie S1. A typical process of directional water self-transport on the silicone oil-infused superhydrophobic surface.

Movie S2. Directional microdroplet transport on the white oil and 1-decanol-infused heterogeneous superwettability surface.

Movie S3. Water droplet dynamic behaviors on the silicone oil-infused superwettability surfaces.

Movie S4. Transporting and collecting microdroplets from different directions.

Movie S5. Continuous driving capability of the silicone oil-infused heterogeneous superwettability surface.

Movie S6. Patterned silicone oil-infused heterogeneous superwettability surface to imitate the popular Pac-Man game.

Movie S7. Water self-transport on the tilted silicone oil-infused heterogeneous superwettability surface.

Movie S8. Anti-gravity water self-transport on the tilted silicone oil-infused heterogeneous superwettability surface.

Movie S9. Chemical microreaction process on the tilted silicone oil-infused heterogeneous superwettability surface.
